# Complete atrioventricular block due to timolol eye drops: a case report and literature review

**DOI:** 10.1186/s40360-019-0370-2

**Published:** 2019-12-02

**Authors:** Zhuoying Wang, Ian Denys, Feng Chen, Lijie Cai, Xuecui Wang, Daniel R. Kapusta, Yongliang Lv, Juan Gao

**Affiliations:** 1 Geriatric Department, The Affiliated Guangji Hospital of Soochow University, Suzhou, Jiang Su Province People’s Republic of China 215137; 2 Department of Pharmacology, Louisiana State University Health Sciences Center New Orelans, New Orleans, LA 70112 USA

**Keywords:** Timolol, Atrioventricular block, Case report

## Abstract

**Background:**

Timolol Maleate is a non-selective beta-adrenergic blocker that is commonly used to treat open-angle glaucoma. Despite its topical administration, ophthalmic timolol enters systemic circulation and produces a systemic beta-adrenergic blockade. We report a case of long-term timolol use that uncovered and worsened an underlying cardiac conduction defect demonstrated as a third degree atrioventricular (AV) block.

**Case presentation:**

A 62-year old male with a 13-year history of glaucoma was hospitalized due to shortness of breath, dizziness, and amaurosis. Electrocardiography indicated a heart rate (HR) of 29 bpm with complete atrioventricular (AV) block, and the HR was significantly increased with the treatment of isoprenaline. However, the patient experienced bradycardic episodes (− 20 Δbpm) immediately after self-administration of timolol eye drops. The AV block and bradycardia resolved 48-h after timolol cessation. The man was discharged 1 week later with an asymptomatic first-degree A-V block. However, he presented with a worsened A-V block at his one-year checkup.

**Conclusion:**

We conclude that chronic topical timolol administration may aggravate a cardiac conduction defect leading to an AV block that is only temporarily resolved by timolol cessation. Patients taking timolol should be routinely monitored for cardiovascular aberrations and if any detected, immediately discontinue timolol therapy. Individuals experiencing timolol induced cardiovascular side effects should receive long term follow-up even if symptoms resolve, as they may be indicative of an underlying conduction defect.

## Background

Topical timolol maleate is a non-selective beta-adrenergic antagonist that is commonly used for the treatment of open-angle glaucoma. Although its mechanism of action is unclear, it may decrease ocular pressure by reducing aqueous humor production [1]. Several studies have reported on topical timolol maleate’s known systemic effects such as bradycardia, hypotension, AV block, worsening congestive heart failure, and inducing bronchospasms [2–5]. However, we report the delayed development of a third-degree AV block in an individual after 13 years of otherwise uneventful timolol therapy, which is the longest latency period reported in literature so far.

## Case presentation

A 62-year old Asian male presented to the emergency department with a 3-day history of intermittent shortness of breath, dizziness, and amaurosis. On examination, the patient appeared distressed and anxious. His temperature was 37 °C**,** HR of 29 bpm, a blood pressure of 190/100 mmHg, and a respiratory rate of 16 breaths per minute with an oxygen saturation of 98%. Medications were hydrochlorothiazide 12.5 mg per day for the last year and 0.5% topical timolol twice daily for 13 years. The 12-lead electrocardiogram (ECG) showed bradycardia with third-degree AV block (Additional file 1: Figure S1). However, electrolytes, troponin T, and n-terminal pro-B-type natriuretic peptide were normal, as well as a normal complete blood count, liver and renal function tests, and echocardiography.

**Fig. 1:**
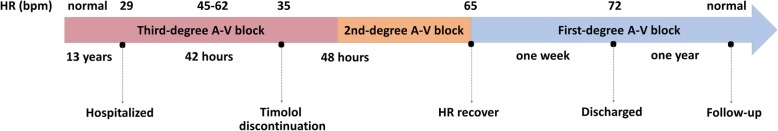
Timeline of Events

Isoprenaline was administered at a dose of 3μg/min and HR increased to 45–62 beats per minute with an ECG showing a third-degree AV block (Additional file 1: Figure S2) with symptom relief. During continuous ECG monitoring we noticed the patient’s HR decreased to 35 bpm 15 min after administering the timolol eye drops. Timolol was replaced by travoprost after consultation with an ophthalmologist. Isoprenaline infusion was continued, but the dose was decreased gradually when HR increased, and stopped when HR recovered to 45–60 bpm at 24-h after the discontinuation of Timolol. The administration of hydrochlorothiazide was continued. Approximately 48 h after discontinuation of timolol, HR of the patient increased to 65 bpm without isoprenaline (Additional file 1: Figure S3), and the ECG showed first-degree AV block with a PR interval of 210 ms. One week after admission, 24-h Holter device monitoring showed an average HR of 72 bpm with an intermittent first-degree AV block. However, the patient did not meet the criteria for pacemaker implantation and was discharged with the addition of 150 mg irbesartan for further blood pressure control. At the 12-month follow-up, Holter recorded first-degree AV block with intermittent second-degree type II AV block and 2:1 AV block at 2:12 AM during the patient’s sleep (Additional file 1: Figure S4). Although this met the criteria of pacemaker implantation, the patient refused it since the AV block was asymptomatic. This patient is currently under continued follow-up care. This case was reported to the State Food and Drug Administration Adverse Event Reporting System, which is the regulatory authority in China. It was not reported to the drug company, due to lack of existing reporting system in this company. The timeline of events is presented in Fig. 1.

## Discussion and conclusion

Since its FDA approval in 1978, timolol has become one of the first line therapies to reduce intraocular pressure in glaucoma. Timolol is a non- selective beta-adrenergic receptor blocker and is generally considered to have few systemic effects when administered opthalmically. However, some research suggests timolol eye drops can reach the nasal mucosa via the nasolacrimal duct leading to roughly 80% of the drug being absorbed into systemic circulation while bypassing the first pass effect [6, 7]. Systemically, timolol can produce beta blocking effects such as bradycardia, conduction block, syncope, and hypotension [8]. Typically, these systemic effects are inconsequential and unheeded in individuals with normal cardiovascular health. However, Lopez showed that in a cohort of 243 patients with a heart block, timolol eye drops were the sole source of the AV block in 12 patients [9]. Startlingly, none of the 12 patients were correctly diagnosed during their first consultation. Tattersall demonstrated that ocular application of beta blockers can markedly reduce the HR of patients with glaucoma, therefore, he suggested that individuals taking ophthalmic timolol should be more closely monitored [10]. Since the cardiovascular side effects are often overlooked yet can be life threatening, we fully support this concept.

We evaluated the causality using the Naranjo scaling method and conclude that the current case is a probable adverse reaction of Timolol (Additional file 1: Table S1), which is confirmed by using the WHO-UMC causality assessment system. These observations strongly suggest that timolol is responsible for transforming the first-degree A-V block to a third-degree A-V block. The pharmacokinetics and pharmacodynamics analysis of the systemic effects of topical timolol demonstrated that the onset of action is in 20–30 min, with a peak effect at 4 h, and a half-life of 4–5 h, and duration of action about 24 h [11]. In the current case, the recovery time was 48 h, which was markedly longer than its pharmacokinetics would suggest. This could be explained by the possibility that this patient is a CYP2D6 slow metabolizer, which would cause timolol to accumulate in the circulation and take longer to be metabolized. Another speculation is that this patient is 62 years old, and the elimination of timolol in an elderly adult is much slower than that in a healthy young adult [12]. Both in vivo and in vitro studies have demonstrated the metabolism of timolol is related to the genetic phenotype of CYP2D6 [13, 14]. Individuals with a slow CYP2D6 metabolism have a higher concentration of timolol in plasma and tend to have more severe adverse effects, compared to fast CYP2D6 metabolizers. Therefore, it is of importance to determine the genetic phenotype of CYP2D6 to avoid the severe adverse effects of timolol on slow CYP2D6 metabolizers. In such patients, the combined uses of timolol and CYP2D6 inhibitors such as fluoxetine, ranitidine, et al. should be prevented.

In the present case report, the AV block appeared after consistent use of timolol for 13 years, which is the longest latency according to literature. We summarized the latency in several case reports searched on PubMed and Google Scholar, which supports the statement that the current report has the longest latency (Table 1) [4, 11, 15–18]. The 13 years of well tolerated timolol use caused physicians to overlook this drug as the causative agent for the AV block. We believe that when this patient started to use timolol at a younger age, his metabolism of this drug was still normal. However, as he was aging, the elimination of timolol was slower [12], and the same dose of administration caused the drug accumulated in the circulation, and eventually led to the complete A-V block. Fortunately, HR recovered 48-h after the discontinuation of timolol and implantation of a pacemaker was not necessary. At the one-year follow-up, an ambulatory EKG recording displayed a paroxysmal second-degree type II AV block and 2:1 AV block. Considering the first-degree A-V block appeared again after a one-year discontinuation of timolol, we reason the patient had a subclinical preexisting conduction defect, that was uncovered and exacerbated by timolol use. We think both timolol and the existing conduction defect synergistically contributed to the complete A-V block in this case. Therefore, it is necessary to have appropriate follow up of patients, who have experienced timolol induced AV block, to detect the reappearance and worsening of a conduction defect.

**Table 1:** General information of current and other cases in literature which had Timolol induced heart block

Patient Age	Patient Gender	Timolol Therapy Duration (year)	Heart Block	Permanent Pacemaker	Follow-up	Reference #
62	M	13	3rd-degree AV block	NO	1 year	Current report
78	F	4.33	3rd-degree AV block	NO	N/A	[15]
85	M	1.25	3rd-degree AV block	YES	N/A	[15]
56	F	3.67	3rd-degree AV block	YES	N/A	[15]
76	M	2.25	3rd-degree AV block	YES	N/A	[15]
80	F	1.00	3rd-degree AV block	YES	N/A	[11]
70	F	30 min prior to heart block onset	AV BLOCK and sick sinus syndrome	YES	N/A	[4]
84	M	0.10	Mobitz I block and sinus dysfunction	NO	2 months	[16]
63	F	A long time	A sinoatrial block	NO	N/A	[17]
88	F	A long time	3rd-degree AV block	NO	6 months	[18]

In conclusion, topical timolol maleate can induce severe systemic effects. Health care providers should be mindful of the potential cardiovascular side effects of timolol and closely monitor patients for the duration of their treatment. In particular, cardiologists should thoroughly review the drug regimen of all patients presenting with an AV block.

## Supplementary information

**Additional file 1: Figure S1.** ECG showed third-degree atrioventricular block when the patient was at admission. The heart rate is 29 bpm. **Figure S2.** ECG showed an improved heart rate after Isoprenaline administered. **Figure S3.** ECG showed a first-degree AV block after timolol was discontinued for 48 h. **Figure S4.** At 1-year follow-up, Holter recorded a I degree AV block with intermittent second-degree type II AV block. The minimum heart rate was 51 bpm with a long R-R interval of 2.16 s. **Table S1.** Adverse Reaction Evaluation Using Naranjo Scale.

## Data Availability

All data generated or analyzed during this study are included in this published article.

## Abbreviations

AV: Atrioventricular
ECG: Electrocardiogram
HR: Heart rate
