# Supplementary material for: Complete atrioventricular block due to timolol eye drops: a case report and literature review
Source: BMC Pharmacol Toxicol. 2019 Dec 2;20:73. doi: 10.1186/s40360-019-0370-2 (PMC6889336; doi:10.1186/s40360-019-0370-2)
Supplement: Supplementary file 1 — Additional file 1: Figure S1. ECG showed third-degree atrioventricular block when the patient was at admission. The heart rate is 29 bpm. Figure S2. ECG showed an improved heart rate after Isoprenaline administered. Figure S3. ECG showed a first-degree AV block after timolol was discontinued for 48 h. Figure S4. At 1-year follow-up, Holter recorded a I degree AV block with intermittent second-degree type II AV block. The minimum heart rate was 51 bpm with a long R-R interval of 2.16 s. Table S1. Adverse Reaction Evaluation Using Naranjo Scale. [file 40360_2019_370_MOESM1_ESM.docx]

**Data Supplement**

**Complete Atrioventricular Block due to Timolol Eye Drops：A Case Report and Literature Review**

Zhuoying Wang, M.D; M.S.^1^, Ian Denys, Ph.D.^2^, Feng Chen, M.D.^1^, Lijie Cai, M.D.^1^, Xuecui Wang, M.D.^1^, Daniel R Kapusta, Ph.D.^2^, Yongliang Lv, M.D.^1,*^, Juan Gao, M.D; M.S.^2,*^

1, Geriatric Department, The Affiliated Guangji Hospital of Soochow University, Suzhou, Jiangsu, PR China;

2, Department of Pharmacology, Louisiana State University Health Sciences Center-New Orleans, LA, USA 70112.

Corresponding authors:

Yongliang Lv, M.D.

Geriatric Department

The Affiliated Guangji Hospital

of Soochow University

Suzhou, Jiang Su Province,

People’s Republic of China, 215137

Tel: +86-512-65797482;

Fax: +86-512-65797482;

E-mail: [lylv@sohu.com](mailto:lylv@sohu.com)

Juan Gao, M.D., M.S.

Department of Pharmacology

Louisiana State University Health

Sciences Center-New Orleans

1901 Perdido Street. MEB5229

New Orleans, LA 70112, USA

Tel: +1-504-568-4780

Fax: +1-504-568-2361

E-mail: jgao1@lsuhsc.edu


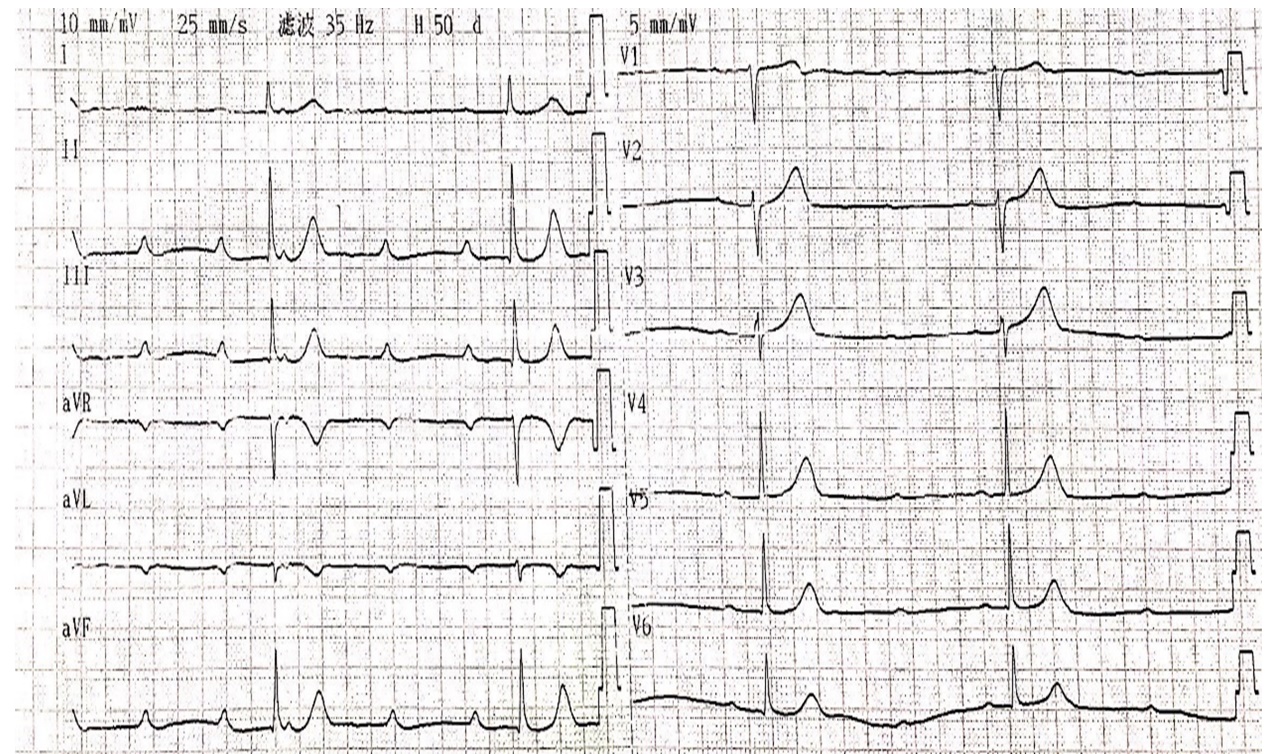


Figure S1. ECG showed third-degree atrioventricular block when the patient was at admission. The heart rate is 29 bpm.


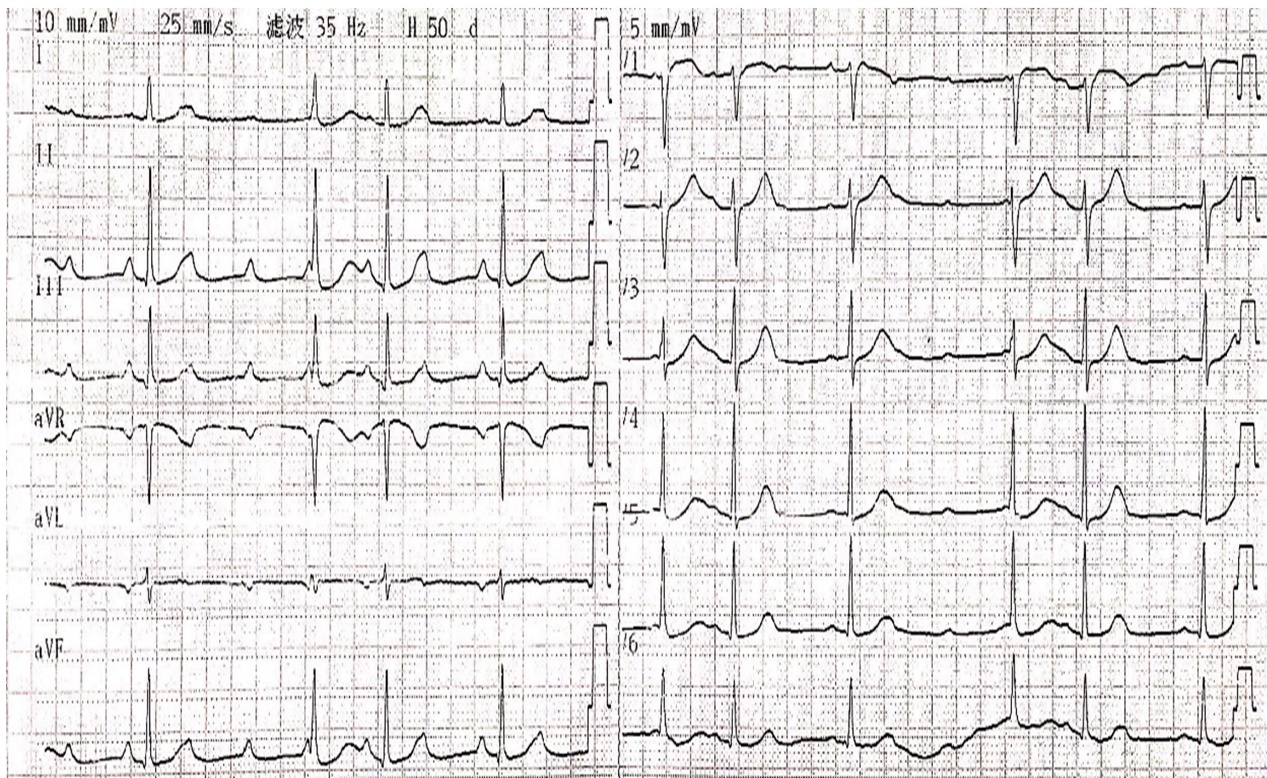


Figure S2. ECG showed an improved heart rate after Isoprenaline administered.


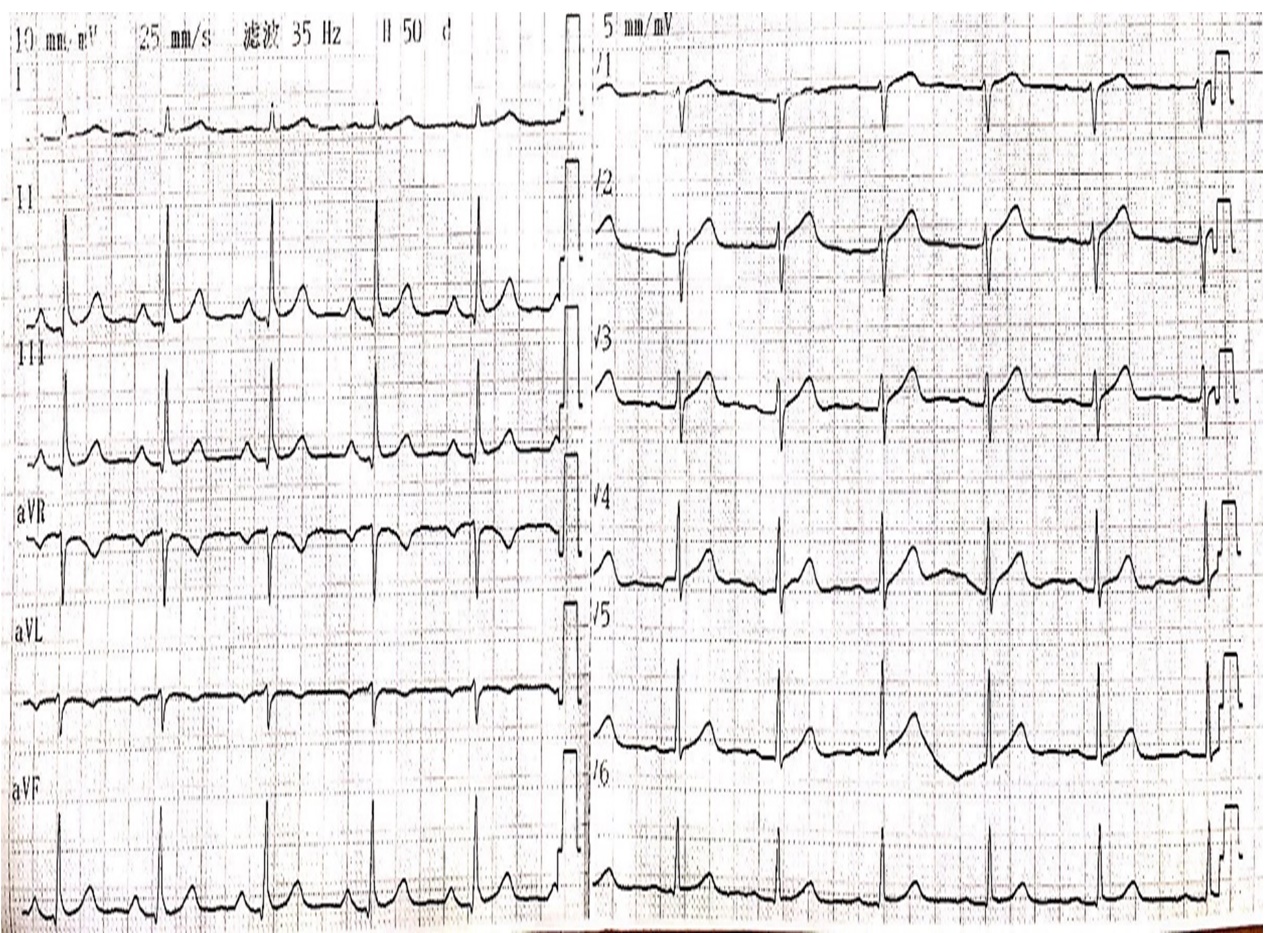


Figure S3. ECG showed a first-degree AV block after timolol was discontinued for 48 hours.


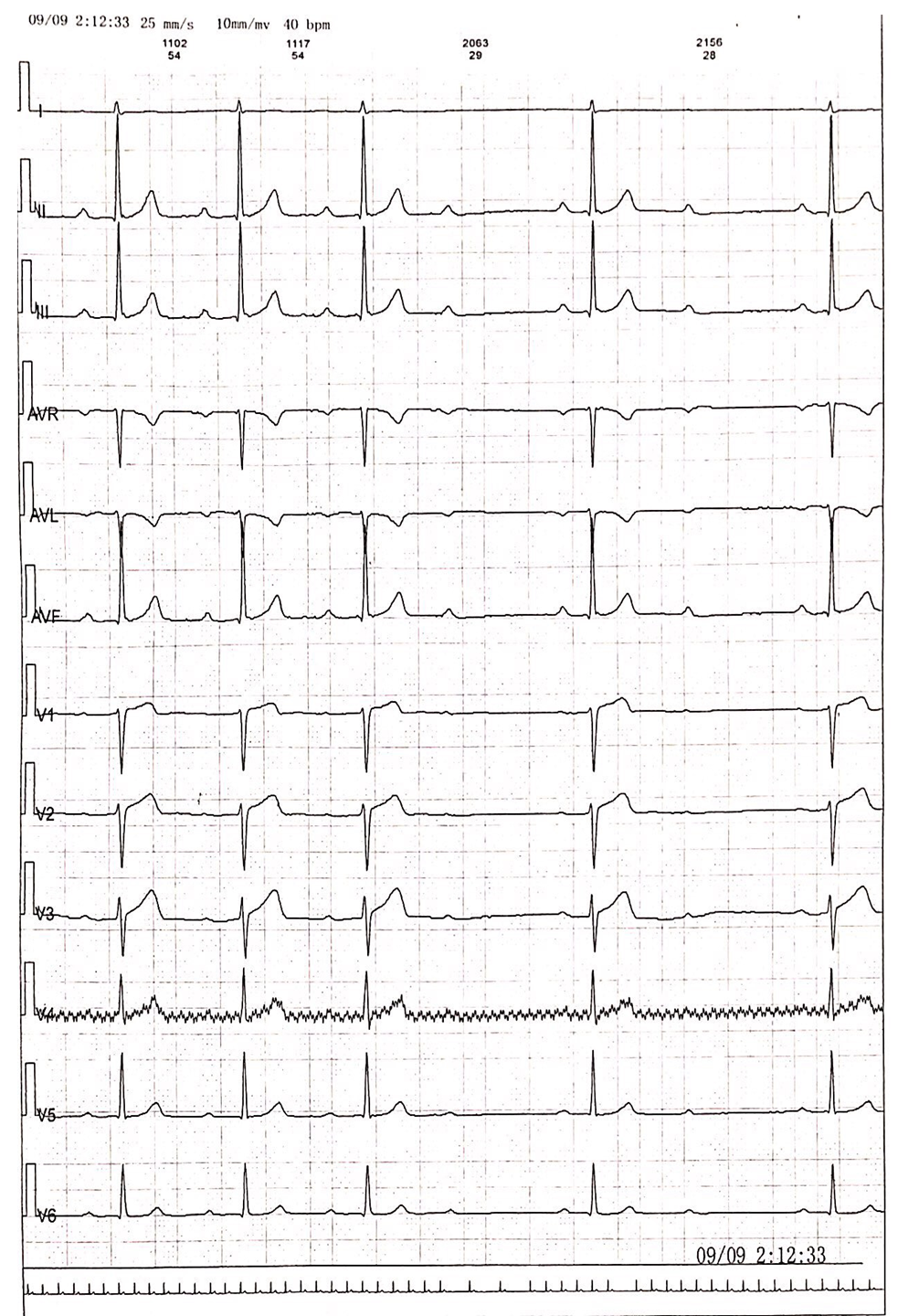


Figure S4. At 1-year follow-up, Holter recorded a I degree AV block with intermittent second-degree type II AV block. The minimum heart rate was 51 bpm with a long R-R interval of 2.16 seconds.

Table S1. Adverse Reaction Evaluation Using Naranjo Scale.

| Items | Yes | No | Do not know or Not done |
| --- | --- | --- | --- |
| 1. Are there previous conclusive reports on this reaction? | +1 |  |  |
| 2. Did the adverse events appear after the suspected drug was given? | +2 |  |  |
| 3. Did the adverse reaction improve when the drug was discontinued or a specific antagonist was given? | +1 |  |  |
| 4. Did the adverse reaction appear when the drug was readministered? | +2* |  |  |
| 5. Are there alternative causes that could have caused the reaction? | -1 |  |  |
| 6. Did the reaction reappear when a placebo was given? |  |  | 0 |
| 7. Was the drug detected in any body fluid in toxic concentrations? |  |  | 0 |
| 8. Was the reaction more severe when the dose was increased, or less severe when the dose was decreased? |  |  | 0 |
| 9. Did the patient have a similar reaction to the same or similar drugs in any previous exposure? |  | 0 |  |
| 10. Was the adverse event confirmed by any objective evidence? | +1 |  |  |

Total score 6. This case is a probable adverse drug reaction (ADR).

*, It is an unintentional rechallenge. Before the patient came to the hospital, he used timolol at home, and then had complete A-V block. After his HR increased and A-V block was attenuated by isoprenaline infusion, he used timolol again by himself, but his HR decreased to 35 bpm 15 minutes later.
